# Global, regional, and national burden of tracheal, bronchus, and lung cancers attributable to high fasting plasma glucose: A systematic analysis of global burden of disease 2019

**DOI:** 10.1111/1753-0407.13499

**Published:** 2023-11-27

**Authors:** Minmin Wang, Jingyi Liu, Jia Wang, Yinzi Jin, Zhi‐Jie Zheng

**Affiliations:** ^1^ Department of Global Health, School of Public Health Peking University Beijing China; ^2^ Institute for Global Health and Development Peking University Beijing China; ^3^ School of Nursing Peking University Beijing China; ^4^ Key Laboratory of Carcinogenesis and Translational Research (Ministry of Education/Beijing) Peking University Cancer Hospital & Institute Beijing China

**Keywords:** global burden of disease, high fasting plasma glucose, tracheal, bronchus, and lung cancer

## Abstract

**Background:**

Tracheal, bronchus, and lung (TBL) cancer is the third most common and lethal type of cancer worldwide. Glucose metabolism disorders, as represented by high fasting plasma glucose (HFPG), increase the risk of development and worsen the prognosis of TBL cancer. This study aimed to evaluate the global disease burden of TBL cancer attributable to HFPG.

**Methods:**

The TBL cancer burden attributable to HFPG was estimated based on a modeling strategy using the Global Burden of Disease Study 2019. The disease burden globally and by regions, countries, development levels, age groups, and sexes were also evaluated with the indicators of death, disability‐adjusted life years, years of life lost, and years lived with disability. The estimated annual percentage change (EAPC) was calculated by regression model to show the temporal trend.

**Results:**

In 2019, approximately 8% of the total TBL cancer burden was attributable to HFPG. The HFPG‐attributable TBL cancer burden increased globally from 1990 to 2019 with the EAPC of 0.98% per year. The burden was positively associated with social development levels, and the global burden was three times greater in men than in women. HFPG‐attributable TBL cancer burden increased with age and peaked at above 70 years of age.

**Conclusions:**

The findings highlight the effect and burden of glucose disorders, as represented by HFPG on TBL cancer burden. Integrated cancer prevention and control measures are needed, with control of glucose disorders as one of the key elements.

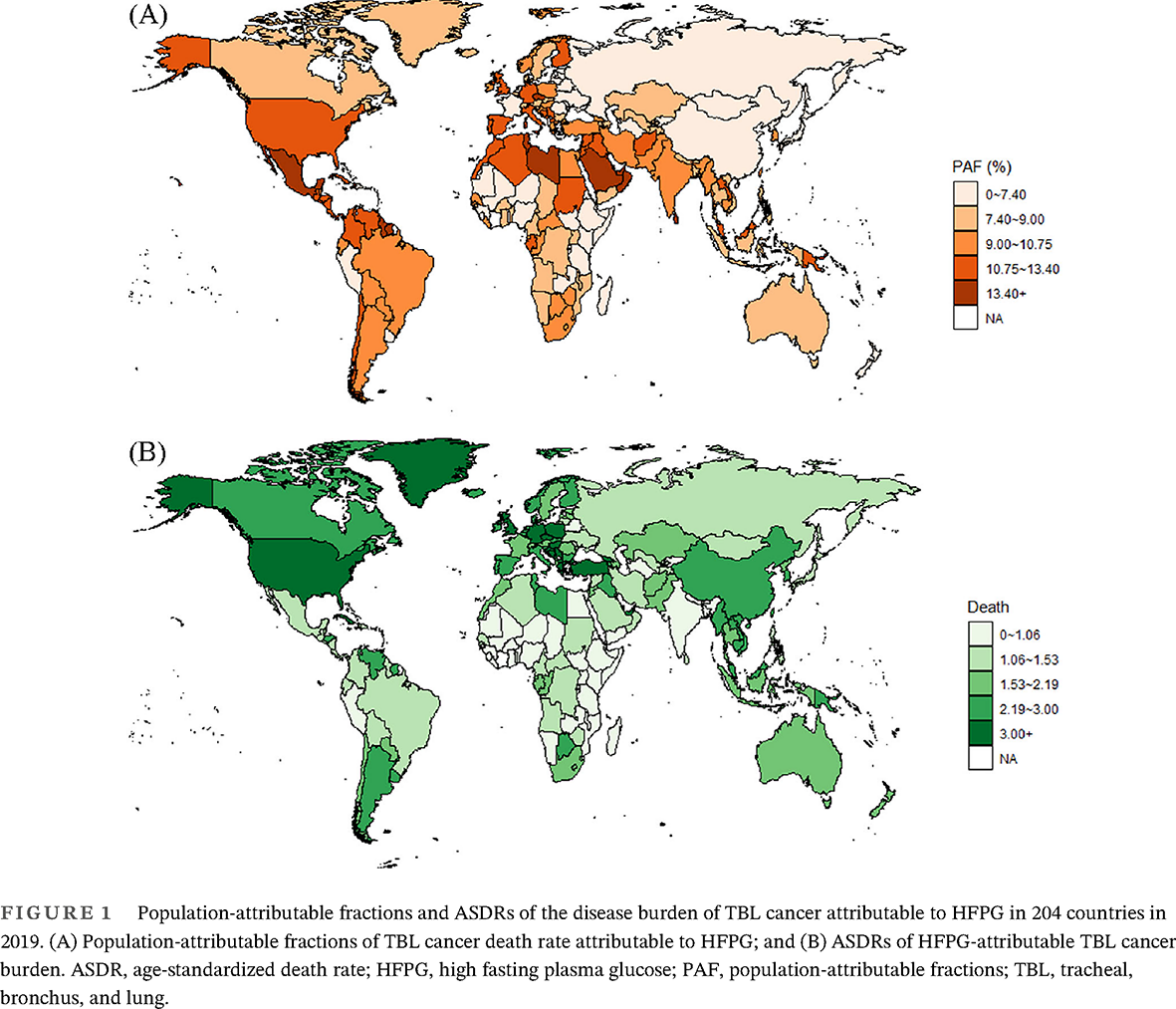

## Graphical abstract

- High fasting plasma glucose (HFPG) increased the risk of development and worsen the prognosis of Tracheal, bronchus, and lung (TBL) cancer, we evaluated the global disease burden of TBL cancer attributable to HFPG.
- HFPG‐attributable TBL cancer burden increased globally from 1990 to 2019, and approximately 8% of TBL cancer burden was attributable to HFPG in 2019.
- Men, people over 70 years of age were vulnerable subgroups in HFPG‐attributable TBL cancer.
- Integrated cancer prevention and control measures are needed, with control of metabolic disorders as one of the key elements.

## 1 INTRODUCTION

Tracheal, bronchus, and lung (TBL) cancer is the third most common cancer and the leading cause of cancer‐related death worldwide.^1^ In 2020, there were 2 206 771 new cases and 1 796 144 deaths worldwide, and the disease burden of TBL cancer showed a rapid growth trend in the decade from 2008 to 2018, with the number of new lung cancer cases increasing by approximately 30% worldwide.^2^, ^3^ It has been estimated that the numbers of new cases and deaths will reach 3 432 024 and 2 909 286, respectively, by 2040, with a doubling of the burden in low development countries and an increase in burden of over 70% in middle and high development countries.^4^ TBL cancer also negatively affects countries' economic growth. An estimation of $3.9 trillion in 2017 international dollars of economic cost would be caused from 2020 to 2050, ranking first among 29 cancer types.^5^ This projection highlights the need to establish prevention strategies to deal with the forthcoming threat from TBL cancers.

Identification of risk factors of TBL cancer by epidemiological studies has facilitated lung cancer prevention strategies. For example, tobacco use is the primary risk factor of lung cancer, and tobacco control has become the key measure for lung cancer prevention.^6^ Recently, glucose disorders were discovered to play a key role in the onset and development of TBL cancer. High fasting plasma glucose (HFPG), a representative indicator of abnormal glucose metabolism, promoted tumor metabolism. One epidemiological study showed that lung cancer incidence increased by 53% and 61% in men and women with diabetes, respectively. A meta‐analysis of observational studies identified a significantly increased risk of lung cancer in diabetes patients after adjusting for tobacco use.^7^ Having a glucose metabolism disorder also had a major impact on the prognosis of lung cancer patients. All‐cause mortality was 1.69 times higher in lung cancer patients with HFPG levels compared with that in patients with normal fasting blood glucose levels.^8^

Although the independent association was identified, lack of estimation of the disease burden as well as population‐attributable fraction of TBL cancer attributable to HFPG would hinder the development of an integrated TBL cancer prevention strategy. In this study, we estimated the global, regional, and national burden and trend of TBL cancer attributable to HFPG to provide evidence for further development of integrated TBL cancer prevention strategies, to indicate the key regions and populations in the strategy implementation, and to provide fundamental data for monitoring intervention progress.

## 2 METHODS

### 2.1 Data sources

The data for disease burden related to HFPG were obtained from the Global Burden of Diseases, Injuries, and Risk Factors Study (GBD) 2019 dataset using the Global Health Data Exchange (https://ghdx.healthdata.org). The Institute for Health Metrics and Evaluation initiated the series of GBD studies in 2002. This institute reported the global, regional, and country‐level disease burden estimation attributable to metabolic, environmental, occupational, and behavioral risk factors based on a comparative risk assessment framework.^9^ In this study, we extracted the disease burden of TBL cancer attributable to HFPG at the global level and regionally for 204 countries and territories from 1990 to 2019.

### 2.2 Definition and estimation framework

Fasting plasma glucose (FPG) was measured as the mean FPG in a population, and high FPG was defined as any level above the theoretical minimum‐risk exposure level, which is 4.8–5.4 mmol/L, as in the GBD 2019 study.^10^ Tracheal cancer, bronchial cancer, and lung cancer refer to all malignant tumors localized in the trachea, bronchi, and lungs, with the International Classification of Diseases, Tenth Revision codes C33, C34‐C34.92, Z12.2 (special screening for respiratory organ tumors), Z80.1‐Z80.2 (family history of malignant tumor in the trachea, bronchi, lung, other respiratory organs, and intrathoracic organs), Z85.1‐Z85.2 (personal history of malignant tumor in the trachea, bronchi, lungs, other respiratory organs, and intrathoracic organs), and the code B.1.11 according to the GBD disease classification code system.^11^

The disease burden (death, disability‐adjusted life years [DALY]; years of life lost [YLL]; and years lived with disability [YLD]) of TBL cancer attributable to HFPG was estimated using the GBD 2019 data set based on a modeling strategy.^12^ The DALY is a health gap measure that extends the idea of potential years of life lost due to premature death, to include equivalent years of “healthy” life lost to poor health or disability. The DALY was mathematically equivalent to the sum of YLL and YLD. To estimate the disease burden, the relative risks of risk‐outcome pairs were summarized based on systematic reviews of published evidence. The exposure of risk factors by age‐sex‐location‐year was then formulated using population survey data with individual‐level information and applying spatiotemporal Gaussian process regression or Bayesian statistical models. Finally, the attributable disease burden was computed for the defined risk‐outcome pairs. The detailed methodologies and input data have been reported elsewhere.^10^

The sociodemographic index (SDI) is a comprehensive indicator based on the education level, per capita income, and total fertility rate of individuals under the age of 25 years, which measures the overall development scale of a country.^13^ The SDI indicator was also extracted from the GBD 2019 data set (https://ghdx.healthdata.org/record/ihme-data/gbd-2019-socio-demographic-index-sdi-1950-2019). In 2019, different countries were classified into five development levels according to the SDI, namely: low (<0.46), low‐middle (0.46–0.60), middle (0.61–0.69), high‐middle (0.70–0.81), and high (>0.81).^13^

### 2.3 Statistical analysis

In this study, all‐age numbers and age‐standardized rate (ASR) of deaths and DALYs of TBL attributable to HFPG were estimated. The disease burden across regions, sexes, and age groups was compared. Estimated average percentage change (EAPC) was computed to depict the secular trend in ASRs of HFPG‐attributable TBL cancer burden based on a regression model by fitting the natural logarithm of ASR with the calendar year, namely, y = α + βx + ɛ, where y = ln (rate), x = calendar year, and ε = error term. In this formula, β represents the positive or negative ASR trends. EAPC and its 95% confidence interval (CI) were calculated from the formula of 100 × (exp (β) −1). The associations between the disease burden of TBL cancer attributable to HFPG and SDI were evaluated by Pearson correlation analysis and visualized by using a scatter plot with a smooth curve. All analysis was conducted using R 4.0.4 and *p* < .05 was considered statistically significant.

## 3 RESULTS

### 3.1 TBL cancer burden attributable to HFPG

In 2019, approximately 8% of the total TBL cancer burden was attributable to HFPG. Globally, HFPG‐attributable TBL cancers caused 179048.78 deaths (95% uncertainty interval [UI]: 42684.98–389384.23), 3640548.35 DALYs (95% UI: 856167.14–8012528.62), 45923.11 years lived with disability (95% UI: 10180.82–104663.00), and 3594625.24 years of life lost (95% UI: 843576.73–7906892.03). The age‐standardized rates of deaths, DALYs, years lived with disability, and years of life lost were 2.22 (95% UI: 0.53–4.83), 44.06 (95% UI: 10.42–96.82), 0.56 (95% UI: 0.12–1.28), and 43.49 (95% UI: 10.26–95.52) per 100 000 population, respectively. From 1990 to 2019, the global burden of TBL attributable to HFPG was stable with a slight increase, EAPC of the age‐standardized rate of death, DALYs, YLLs, and YLDs reached 0.98 (95% CI: 0.82–1.15), 0.68 (95% CI: 0.55–0.82), 0.68 (95% CI: 0.54–0.81), and 1.32 (95% CI: 1.15–1.49), separately (Tables 1, S1 and S2).

**TABLE 1:** Age‐standardized death rate and population‐attributable fractions of TBL cancer attributable to HFPG in 2019, by regions and sexes.

Regions	Both		Women		Men	
	ASDR	PAF of death (%)	ASDR	PAF of death (%)	ASDR	PAF of death (%)
Global	2.22 (0.53–4.83)	8.84 (2.05–19.21)	1.25 (0.25–2.93)	8.35 (1.56–19.18)	3.42 (0.58–7.88)	9.15 (1.55–20.91)
East Asia	2.72 (0.61–6.19)	7.10 (1.58–15.82)	1.54 (0.29–3.78)	6.78 (1.21–16.18)	4.20 (0.7–10.16)	7.31 (1.19–17.20)
Southeast Asia	1.99 (0.45–4.50)	8.65 (2.03–18.81)	1.14 (0.23–2.79)	8.52 (1.63–19.52)	3.10 (0.52–7.44)	8.86 (1.49,20.38)
Oceania	3.10 (0.66–7.06)	13.57 (3.01–28.64)	1.27 (0.26–3.06)	12.72 (2.66–28.11)	4.92 (0.88–11.62)	13.79 (2.50–30.38)
Central Asia	1.61 (0.35–3.56)	8.46 (1.86–18.65)	0.64 (0.13–1.51)	8.53 (1.64–19.8)	2.98 (0.49–6.90)	8.51 (1.44–19.85)
Central Europe	3.83 (0.83–8.39)	10.02 (2.33–21.79)	1.70 (0.33–3.95)	8.68 (1.71–20.06)	6.65 (1.14–15.21)	10.69 (1.88–24.11)
Eastern Europe	1.14 (0.23–2.68)	5.04 (0.99–11.67)	0.36 (0.06–0.86)	4.90 (0.91–11.80)	2.47 (0.41–6.01)	5.22 (0.83–12.66)
High‐income Asia Pacific	1.72 (0.36–3.87)	7.75 (1.70–17.30)	0.69 (0.13–1.66)	6.03 (1.09–14.50)	3.07 (0.50–7.16)	8.49 (1.40–19.92)
Australasia	1.86 (0.43–4.09)	7.84 (1.82–17.06)	1.25 (0.24–3.01)	6.74 (1.31–16.16)	2.59 (0.45–6.06)	8.66 (1.47–19.93)
Western Europe	2.90 (0.69–6.21)	9.99 (2.35–21.39)	1.61 (0.31–3.72)	9.02 (1.75–20.72)	4.48 (0.77–10.11)	10.53 (1.84–23.53)
Southern Latin America	2.23 (0.52–4.80)	9.50 (2.22–20.50)	1.24 (0.24–2.83)	8.88 (1.76–20.16)	3.52 (0.60–8.04)	9.87 (1.72–22.18)
High‐income North America	4.54 (1.12–9.46)	12.64 (3.12–26.11)	3.25 (0.67–7.37)	10.99 (2.15–24.84)	6.12 (1.1–13.24)	14.01 (2.53–30.59)
Caribbean	2.55 (0.59–5.52)	11.78 (2.82–24.87)	1.47 (0.30–3.46)	10.37 (2–23.41.00)	3.82 (0.69–8.52)	12.61 (2.28–27.75)
Andean Latin America	0.96 (0.22–2.18)	8.45 (1.99–18.26)	0.79 (0.16–1.89)	8.14 (1.55–18.87)	1.15 (0.19–2.75)	8.7 (1.47–19.96)
Central Latin America	1.53 (0.37–3.29)	12.93 (3.15–26.95)	1.03 (0.21–2.38)	11.96 (2.40–26.53)	2.11 (0.37–4.76)	13.58 (2.48–29.36)
Tropical Latin America	1.45 (0.34–3.12)	9.14 (2.17–19.75)	0.99 (0.19–2.30)	8.47 (1.63–19.59)	2.04 (0.34–4.64)	9.66 (1.64–22.04)
North Africa and Middle East	1.91 (0.41–4.17)	10.91 (2.47–23.65)	0.83 (0.17–1.95)	10.83 (2.17–24.18)	2.97 (0.49–6.86)	10.96 (1.90–24.86)
South Asia	0.86 (0.19–1.92)	9.81 (2.25–21.45)	0.43 (0.08–1.03)	9.10 (1.84–21.07)	1.31 (0.23–3.09)	10.10 (1.73–23.28)
Central Sub‐Saharan Africa	1.22 (0.23–3.46)	9.03 (1.95–19.86)	0.42 (0.08–1.03)	7.01 (1.34–16.61)	2.31 (0.35–7.32)	9.95 (1.67–22.78)
Eastern Sub‐Saharan Africa	0.49 (0.10–1.12)	6.40 (1.35–14.51)	0.20 (0.04–0.49)	5.23 (0.97–12.61)	0.82 (0.13–1.96)	6.90 (1.16–16.45)
Southern Sub‐Saharan Africa	1.89 (0.45–4.03)	9.86 (2.38–21.03)	1.20 (0.25–2.73)	10.74 (2.09–24.14)	2.95 (0.50,6.71)	9.58 (1.63–21.97)
Western Sub‐Saharan Africa	0.72 (0.16–1.65)	7.27 (1.58–16.07)	0.36 (0.07–0.86)	6.91 (1.32–16.21)	1.12 (0.18–2.70)	7.41 (1.22–17.44)

Abbreviations: ASDR, age‐standardized death rate; HFPG, high fasting plasma glucose; PAF, population‐attributable fractions; TBL, tracheal, bronchus, and lung.

### 3.2 TBL cancer burden attributable to HFPG by regions and countries

In 2019, the TBL cancer burden attributable to HFPG displayed disparity across regions. High‐income North America had the highest age‐standardized death rate of 4.54 (95% UI: 1.12–9.46) per 100 000 population, and Eastern Europe had the lowest age‐standardized death rate of 0.49 (95% UI: 0.10–1.12) per 100 000 population. The highest population‐attributable fraction (PAF) of TBL cancer death attributable to HFPG was identified in Oceania at 13.57% (95% UI: 3.01%–28.64%). The lowest PAF of TBL cancer death attributable to HFPG was identified in Eastern Europe at 5.04% (95% UI: 0.99%–11.67%).

**FIGURE 1:**
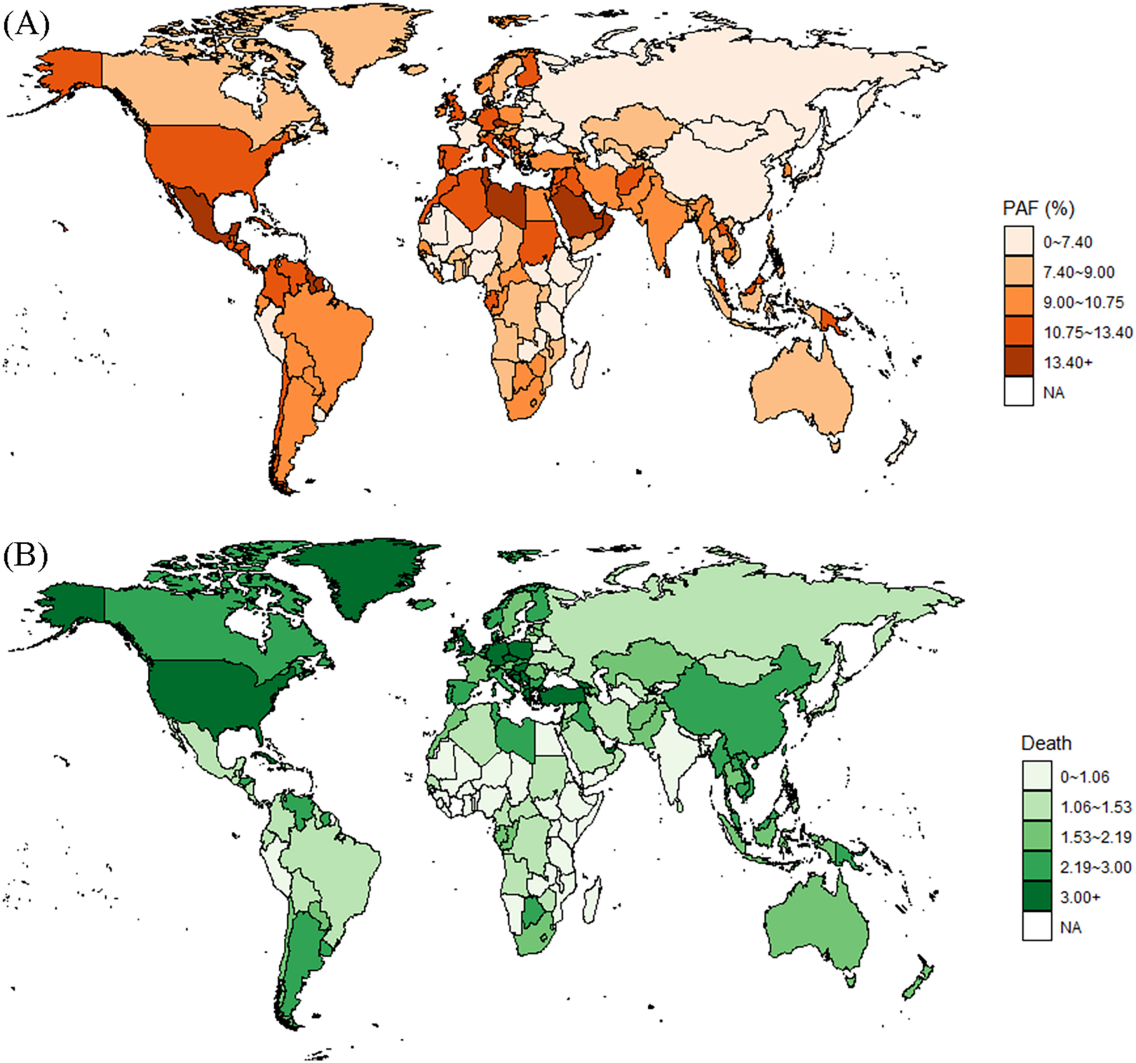
Population‐attributable fractions and ASDRs of the disease burden of TBL cancer attributable to HFPG in 204 countries in 2019. (A) Population‐attributable fractions of TBL cancer death rate attributable to HFPG; and (B) ASDRs of HFPG‐attributable TBL cancer burden. ASDR, age‐standardized death rate; HFPG, high fasting plasma glucose; PAF, population‐attributable fractions; TBL, tracheal, bronchus, and lung.

The HFPG‐attributable TBL cancer burden also showed disparity at the country level (Figure 1). In 2019, Brunei Darussalam had the highest age‐standardized death rate of 8.77 (95% UI: 2.39–17.42) per 100 000 population and Ethiopia had the lowest age‐standardized death rate of 0.34 (95% UI: 0.06–0.85) per 100 000 population. The highest PAF of TBL cancer death attributable to HFPG was identified in Qatar at 24.50% (95% UI: 5.99%–47.34%). The lowest PAF of TBL cancer death attributable to HFPG was identified in Mongolia at 4.07% (95% UI: 0.85%–9.57%).

### 3.3 TBL cancer burden attributable to HFPG by SDI regions

**FIGURE 2:**
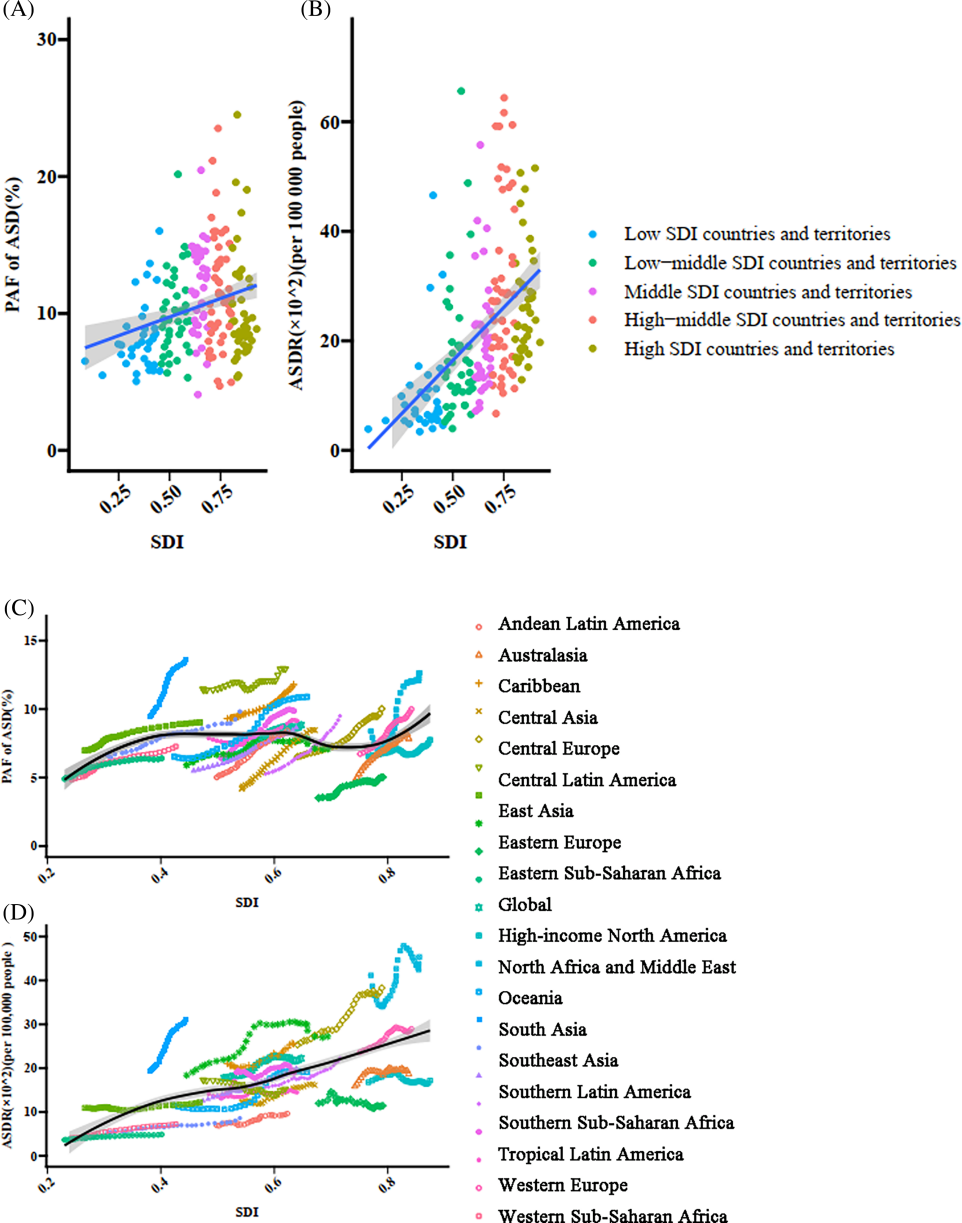
The correlation between population‐attributable fractions and ASDRs of the disease burden of TBL cancer attributable to HFPG and SDI levels. (A) The correlation between population‐attributable fractions of TBL cancer death rate attributable to HFPG and SDI levels in 204 countries in 2019. (B) The correlation between ASDRs of TBL cancer death rate attributable to HFPG and SDI levels in 204 countries in 2019. (C) The correlation between population‐attributable fractions of TBL cancer death rate attributable to HFPG and SDI levels in 21 regions from 1990 to 2019. (D) The correlation between ASDRs of TBL cancer death rate attributable to HFPG and SDI levels in 21 regions from 1990 to 2019. ASDR, age‐standardized death rate; HFPG, high fasting plasma glucose; PAF, population‐attributable fractions; SDI, sociodemographic index; TBL, tracheal, bronchus, and lung.

The disease burden of TBL cancer attributable to HFPG was highly correlated with the regional development level. In regions with high and high‐middle SDIs, the TBL cancer burden attributable to HFPG was higher than the global average. The correlation between TBL cancer burden attributable to HFPG and the SDI index is shown in Figure 2A,B. Increased PAF (r = 0.26, *p* < .001) and age‐standardized death rate (r = 0.47, *p* < .001) were correlated with increasing SDI index. From 1990 to 2019, the PAF of TBL cancer attributable to HFPG increased in each SDI region. The age‐standardized death rate increased slightly in high SDI regions with EAPC of 0.69% (95% CI: 0.55%–0.84%), and the largest increment appeared in low‐middle SDI regions with EAPC of 1.68% (95% CI: 1.47%–1.90%) (Table 2).

**TABLE 2:** Disease burden of TBL cancer attributable to HFPG from 1990 to 2019, by SDI regions and sexes.

Category	Age‐standardized rate of Death		Age‐standardized rate of DALY		Age‐standardized rate of YLD		Age‐standardized rate of YLL	
	1990	2019	1990	2019	1990	2019	1990	2019
Both								
Global	1.78 (0.39–3.99)	2.22 (0.53–4.83)	37.84 (8.10–85.68)	44.06 (10.42–96.82)	0.41 (0.09–0.97)	0.56 (0.12–1.28)	37.43 (8.02–84.83)	43.49 (10.26–95.52)
High SDI	2.76 (0.62–6.07)	3.11 (0.76–6.60)	59.41 (13.15–131.41)	61.28 (14.74–131.02)	0.73 (0.16–1.68)	0.94 (0.22–2.13)	58.68 (13.01–130.03)	60.34 (14.53–129.02)
High‐middle SDI	1.92 (0.40–4.40)	2.41 (0.55–5.32)	43.34 (8.77–100.46)	49.47 (10.88–110.18)	0.42 (0.09–1.01)	0.59 (0.13–1.38)	42.92 (8.69–99.45)	48.87 (10.75–109.10)
Middle SDI	1.35 (0.30–3.08)	2.10 (0.48–4.68)	28.78 (6.26–66.52)	41.82 (9.52–94.98)	0.27 (0.06–0.66)	0.47 (0.10–1.10)	28.51 (6.19–65.85)	41.35 (9.42–94.05)
Low‐middle SDI	0.71 (0.15–1.65)	1.17 (0.27–2.61)	15.12 (3.04–35.22)	24.57 (5.43–55.45)	0.14 (0.03–0.35)	0.24 (0.05–0.57)	14.98 (3.01–34.93)	24.32 (5.37–54.71)
Low SDI	0.54 (0.10–1.33)	0.79 (0.17–1.81)	11.13 (2.05–27.76)	16.64 (3.48–38.41)	0.11 (0.02–0.27)	0.16 (0.03–0.39)	11.03 (2.03–27.50)	16.48 (3.43–38.17)
Women								
Global	0.83 (0.16–1.98)	1.25 (0.25–2.93)	17.35 (3.21–41.45)	24.42 (4.82–58.24)	0.20 (0.04–0.49)	0.33 (0.06–0.79)	17.16 (3.18–40.99)	24.10 (4.77–57.50)
High SDI	1.30 (0.25–3.06)	1.99 (0.40–4.59)	28.11 (5.28–66.15)	39.59 (7.87–91.19)	0.36 (0.06–0.87)	0.63 (0.11–1.50)	27.75 (5.21–65.31)	38.96 (7.77–89.67)
High‐middle SDI	0.73 (0.14–1.77)	1.15 (0.22–2.74)	15.74 (2.94–38.34)	23.40 (4.45–56.19)	0.16 (0.03–0.40)	0.29 (0.06–0.71)	15.58 (2.91–37.93)	23.11 (4.40–55.65)
Middle SDI	0.78 (0.15–1.86)	1.19 (0.23–2.88)	16.33 (3.11–39.72)	23.18 (4.46–56.45)	0.16 (0.03–0.40)	0.27 (0.05–0.67)	16.17 (3.08–39.29)	22.92 (4.41–55.67)
Low‐middle SDI	0.32 (0.06–0.78)	0.64 (0.13–1.53)	6.71 (1.25–16.25)	13.12 (2.68–31.67)	0.07 (0.01–0.17)	0.14 (0.03–0.34)	6.65 (1.24–16.07)	12.98 (2.65–31.39)
Low SDI	0.16 (0.03–0.39)	0.34 (0.07–0.81)	3.31 (0.60–8.19)	7.17 (1.42–17.12)	0.03 (0.01–0.09)	0.07 (0.01–0.19)	3.28 (0.60–8.10)	7.10 (1.41–16.93)
Men								
Global	3.02 (0.49–7.16)	3.42 (0.58–7.88)	62.41 (10.14–149.22)	66.58 (11.24–153.36)	0.68 (0.11–1.65)	0.84 (0.14–2.00)	61.73 (10.02–147.64)	65.73 (11.10–151.28)
High SDI	4.86 (0.8–11.26)	4.51 (0.78–10.10)	100.56 (16.32–235.60)	86.40 (14.79–195.70)	1.23 (0.20–2.97)	1.33 (0.23–3.15)	99.33 (16.14–232.68)	85.07 (14.56–192.85)
High‐middle SDI	3.62 (0.59–8.67)	4.06 (0.67–9.34)	79.36 (12.78–191.95)	81.11 (13.30–189.82)	0.78 (0.12–1.93)	0.98 (0.16–2.33)	78.58 (12.64–190.12)	80.13 (13.13–187.52)
Middle SDI	2.04 (0.32–4.87)	3.16 (0.51–7.38)	42.43 (6.59–101.85)	62.52 (10.12–146.39)	0.41 (0.06–1.01)	0.70 (0.11–1.70)	42.03 (6.53–100.80)	61.82 (10.01–145.00)
Low‐middle SDI	1.11 (0.17–2.69)	1.77 (0.31–4.09)	23.45 (3.61–57.79)	37.07 (6.41–86.13)	0.22 (0.03–0.55)	0.36 (0.06–0.88)	23.23 (3.57–57.25)	36.70 (6.34–85.48)
Low SDI	0.91 (0.14–2.34)	1.28 (0.22–3.09)	18.72 (2.93–48.72)	26.53 (4.48–65.06)	0.18 (0.03–0.48)	0.25 (0.04–0.63)	18.55 (2.90–48.23)	26.27 (4.44–64.44)

Abbreviations: DALYs, disability‐adjusted life years; HFPG, high fasting plasma glucose; SDI, sociodemographic index; TBL, tracheal, bronchus, and lung; YLD, years lived with disability; YLLs, years of life lost.

### 3.4 TBL cancer burden attributable to HFPG by sex

In 2019, the burden of TBL tumors attributable to HFPG in men was approximately three times that in women globally (Table 1). The age‐standardized death rate of TBL cancer attributable to HFPG was 3.42 (95% UI: 0.58–7.88) per 100 000 population in men and 1.25 (95% UI: 0.25–2.93) per 100 000 population in women. From 1990 to 2019, the changing pattern of HFPG‐attributable TBL cancer burden was different across SDI regions in men and women. The TBL cancer burden attributable to HFPG in men decreased in high SDI regions, increased and then decreased in high‐middle SDI regions, whereas it increased in other regions (Figure S1). By contrast, the burden of TBL cancer in women attributable to HFPG increased globally with 50.60% growth from 1990 to 2019. This pattern was consistent in all SDI regions for women.

### 3.5 TBL cancer burden attributable to HFPG by age groups

**FIGURE 3:**
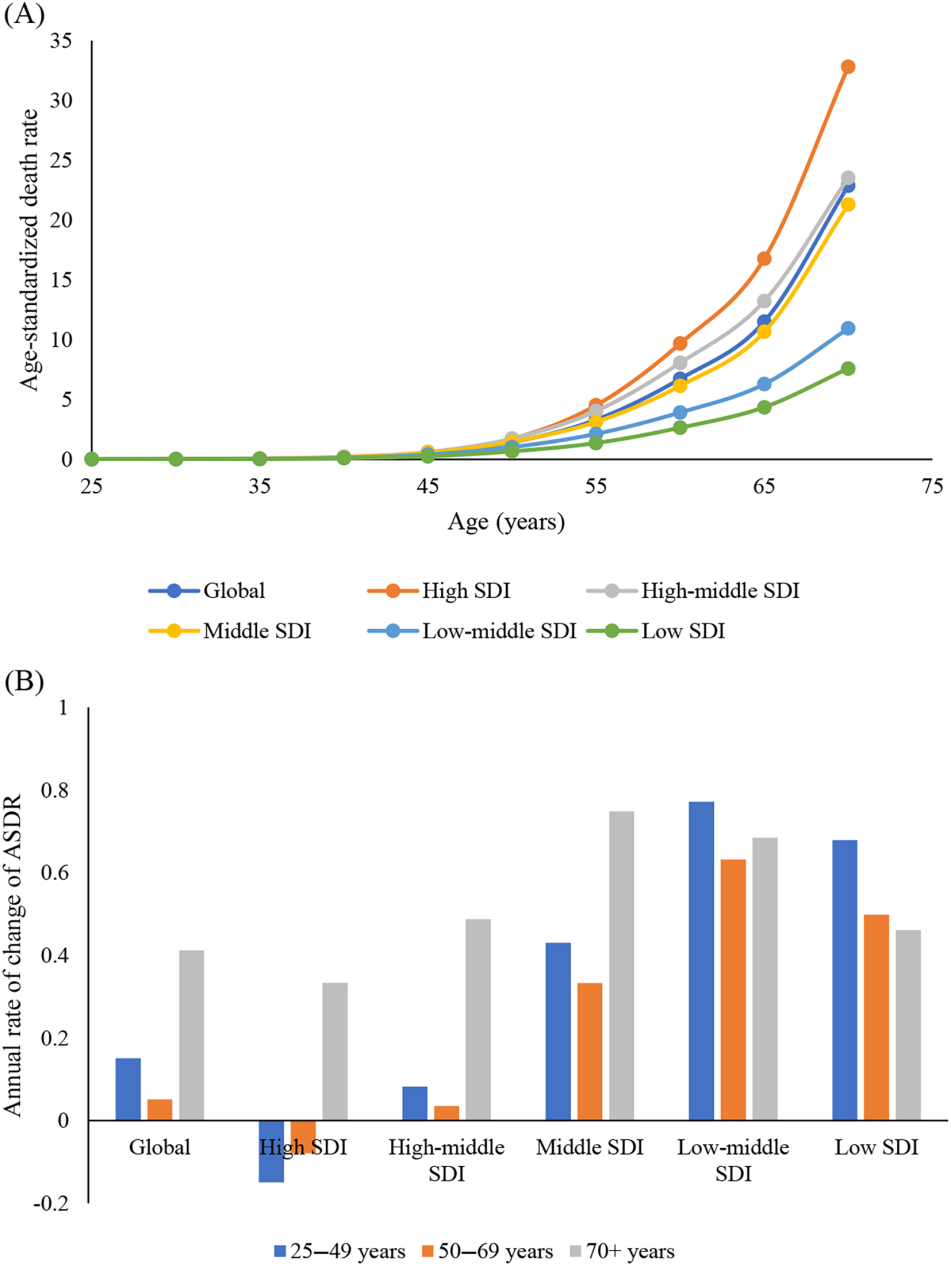
Age trends of the ASDRs of HFPG‐attributable TBL cancer. (A) Age trend of ASDRs of HFPG‐attributable TBL cancer in 2019 by SDI regions. (B) Annual percentage change in ASDRs for TBL cancers attributable to HFPG in three age groups from 1990 to 2019 by SDI regions. ASDR, age‐standardized death rate; HFPG, high fasting plasma glucose; SDI, sociodemographic index; TBL, tracheal, bronchus, and lung.

The disease burden of TBL tumors attributable to HFPG increased with age (Figure 3A). From 1990 to 2019, the TBL cancer burden attributable to HFPG significantly increased across all SDI regions for the age group 70 years and above. However, the TBL cancer burden attributable to HFPG in individuals aged 25–49 years and 50–69 years decreased in the high SDI region, remained stable in the high‐middle SDI region, and significantly increased in middle, low‐middle, and low‐middle and low SDI regions (Figure 3B).

## 4 DISCUSSION

Globally, lung cancer is the malignant tumor with the highest incidence and mortality. Metabolic disorders represented by HFPG are closely related to the onset and development of lung cancer. In this study, we systematically estimated the global, regional, and national TBL cancer burden and trends attributable to HFPG and evaluated the disparity across age groups and sexes. Results indicated that 8% of the total TBL cancer burden was attributable to HFPG, and regions with higher development levels had an increased HFPG‐related TBL cancer burden. The TBL cancer burden attributable to HFPG in men was approximately three times that in women and the burden increased with age. Quantification of the HFPG‐attributable TBL cancer burden could provide epidemiological evidence in formulating integrated TBL cancer prevention strategy as well as indicating which vulnerable regions and populations should be paid attention in strategy implementation.

Evidence has accumulated on the association between HFPG and TBL cancer, especially lung cancer. The Warburg effect postulates that tumor cells consume the majority of glucose by fermentation to lactate rather than by oxidization, even when oxygen is abundant.^14^, ^15^, ^16^ This observation is a hallmark of proliferative metabolism, which is associated with cancer development and progression. The metabolic phenotype of lung cancer cells is characterized by increased glucose uptake and glycolytic activity, which is an independent predictor of survival in patients with non‐small cell lung cancer.^17^ A hyperglycemia‐induced increase in oxidative stress is involved in the acceleration of tumor metastasis, and the removal of systemic hydrogen peroxide by glycol‐conjugated catalase can inhibit the progression of diabetic conditions and tumor metastasis in mice with diabetes.^18^ An epidemiological study with 19 years of follow‐up in 2438 Japanese participants aged 40–79 years reported that the risk of cancer death was significantly higher in individuals with FPG levels of ≥5.6 mmol/L, revealing the role of HFPG in lung cancer etiology.^19^ Moreover, HFPG is also associated with poor overall survival among women with lung cancer.^20^ Results of the study estimated that approximately 8% of the total TBL cancer burden was attributable to HFPG, making HFPG the fourth leading cause of TBL cancer, following the contribution of tobacco (66.36%), air pollution (18.86%), and occupational risk (14.37%). Then it made glucose metabolism a potential intervention target for TBL cancer prevention, especially in high‐income North America, Central Latin America, and Oceania where over one‐sixth of the burden of TBL cancer was attributable to HFPG.

The proportion of TBL cancer attributable to HFPG, as well as the HFPG‐attributable TBL burden, was positively correlated with the level of social development. This observation was also identified in the global estimates of HFPG‐attributed total diseases burden.^21^, ^22^ The reasons for the regional disparity may be explained by social transition and the high prevalence of metabolic disorders in highly developed countries. A literature review reported that diabetes prevalence was higher in high‐income countries than middle and low‐income countries.^23^, ^24^ The association was also identified in another global estimation, and a rising prevalence of diabetes was forecasted for lower‐income countries. In this study, we also found that TBL cancer burden attributable to HFPG increased the most in low‐middle SDI regions, which may be because of a rapid economic and social transition to unhealthy lifestyles and dietary habits. Furthermore, deficits in the quality and accessibility of medical services have resulted in an increased disease burden of type 2 diabetes, which in turn has led to a continuous increase in the disease burden of TBL cancers attributable to HFPG in these regions.

In this study, the TBL cancer burden attributable to HFPG was three times greater in men than in women. Several possible reasons existed for the sex difference. First, the overall TBL cancer burden in men was double that in women in the global cancer registry study.^1^ In 2020, the age‐standardized mortality rate was 25.9 per 100 000 population in men and 11.2 per 100 000 population in women. Second, the prevalence of glucose disorders was slightly more common in men than women, as demonstrated in a global estimate that 17 million more men than women had diabetes in 2017.^25^, ^26^ Third, the association between HFPG and TBL cancer prognosis was significantly stronger in men. A Chinese longitudinal cohort study indicated that men with HFPG had a greater risk of cancer deaths than women, which was hypothesized to be related to the protective effect of estrogen.^27^ Nevertheless, the rapidly growing disease burden in women raises concerns. Studies have shown that the global age‐standardized average HFPG level has increased by 0.07 mmol/L in men and by 0.09 mmol/L in women per decade.^28^

This study indicated that the burden of TBL cancer attributable to HFPG is concentrated in people >50 years of age, with a peak in the age group >70 years. Glucose disorders are common in middle‐aged and older individuals. For example, the prevalence of diabetes reached 24.7% in American adults aged 65 years or older compared with a prevalence of 16.2% in adults aged 45–64 years.^29^ From 1980 to 2012, the prevalence of diabetes continuously increased in the age groups 45–64 years and 65–79 years, aggravating the concerning situation in the elderly.^30^ Variation of glucose metabolism also contributed to the oncogenesis of lung cancer as reported by epidemiological studies. A high variability in fasting blood glucose, systolic blood pressure, total cholesterol, and body weight were each associated with an increased risk of lung cancer.^31^ Thus, the control of glucose disorders in middle‐aged individuals may be a strategy for lung cancer prevention and control especially for elderly individuals >70 years of age.

Evidence from the current study has practical implications for development of the TBL cancer prevention strategy for policy makers. First, results of this study suggested that approximately 8% of the total TBL cancer burden was attributable to HFPG, making HFPG the fourth leading cause of TBL cancer, suggesting that control and management of glucose disorders could be integrated into the TBL cancer prevention strategy. The spatial and temporal distribution of HFPG‐attributable TBL cancer burden suggested that high development countries were of high burden and low‐middle SDI regions had the highest increment, which should be prioritized in glucose disorder management for TBL cancer prevention. At the country level, men and the elderly who bear a higher burden should receive more attention. Overall, results of this study provide the fundamental data to inform the development of an integrated TBL cancer prevention strategy to identify the priority population in strategy implementation.

This study had certain advantages. We systematically estimated the global burden of TBL cancer attributable to HFPG at global, regional, and country levels to inform further global strategy and to provide the fundamental data for monitoring the intervention progress. This study also had limitations. First, several risk factors such as tobacco, air pollution, and obesity were not fully adjusted in the association analysis between SDI level and HFPG‐attributable TBL cancer burden. And the level of plasma glucose was not defined in response to the risk of TBL cancer. Second, the outcome disease was defined as the summary of TBL cancer, but the disease burden by cancer location or histological subtypes was not reported. Third, the methodology of this estimation was based on meta‐analysis of a previously reported study, so interpretation of the study results should be with caution in certain areas with poor data quality.

## 5 CONCLUSIONS

In conclusion, the HFPG‐attributable TBL cancer burden has increased globally from 1990 to 2019. The burden is positively associated with social development levels and is approximately three times greater in men than in women. The HFPG‐attributable TBL cancer burden increases with increasing age and peaks at >70 years of age. The findings highlight the effect and burden of metabolic disorders, as represented by HFPG‐attributable TBL cancer burden. These results call for integrated cancer prevention and control measures, with control of metabolic disorders as one of the key elements.

## AUTHOR CONTRIBUTIONS

All authors helped develop the study concept and design. Minmin Wang: conceptualization, methodology, writing‐ original draft preparation; Jingyi Liu: data curation, writing—original draft preparation; Jia Wang: validation; Yinzi Jin: writing—supervision reviewing and editing, funding acquisition; Zhi‐Jie Zheng: supervision. All authors revised the manuscript and approved the final version.

## FUNDING INFORMATION

This study was funded by the Bill and Melinda Gates Foundation (No. INV‐0045085) and China Postdoctoral Science Foundation (2023M730112). The study sponsor had no role in the study design, data analysis and interpretation of data, the writing of the manuscript, or the decision to submit the paper for publication.

## DISCLOSURE

The authors declare that they have no competing interests.

## CONSENT FOR PUBLICATION

Not applicable.

## Supporting information


**Data S1:** Supporting information

## Data Availability

The datasets generated and/or analyzed during the current study are available in the Global Health Data Exchange (http://ghdx.healthdata.org).
