# Supplementary material for: Global, regional, and national burden of tracheal, bronchus, and lung cancers attributable to high fasting plasma glucose: A systematic analysis of global burden of disease 2019
Source: J Diabetes. 2023 Nov 27;16(3):e13499. doi: 10.1111/1753-0407.13499 (PMC10925880; doi:10.1111/1753-0407.13499)
Supplement: Supplementary file 1 — Data S1: Supporting information [file JDB-16-e13499-s001.docx]

**Global, regional and national burden of tracheal, bronchus, and lung cancer attributable to high fasting plasma glucose: a systematic analysis of Global Burden of Disease 2019**

**Supplemental Materials**

Supplemental Table 1. The disease burden of tracheal, bronchus, and lung cancer attributable to high fasting plasma glucose in 1990 and 2019, by genders and SDI levels.

Supplemental Table 2. The population-attributable fractions of tracheal, bronchus, and lung cancer attributable to high fasting plasma glucose in 1990 and 2019, by genders and SDI levels.

Supplemental Figure 1. Population-attributable fractions and ASDRs for the disease burden of tracheal, bronchial and lung cancers attributable to HFPG in globally and in SDI regions from 1990 to 2019.

**Supplemental Table 1.** The disease burden of tracheal, bronchus, and lung cancer attributable to high fasting plasma glucose in 1990 and 2019, by sexes and SDI levels.

|  | All-age numbers of Death | | All-age numbers of DALY | | All-age numbers of YLD | | All-age numbers of YLL | |
| --- | --- | --- | --- | --- | --- | --- | --- | --- |
|  | 1990 | 2019 | 1990 | 2019 | 1990 | 2019 | 1990 | 2019 |
| **Both** | | | | | | | | |
| Global | 67263.73 (14644.6,151217.80) | 179048.78 (42684.98,389384.23) | 1510452.26 (321413.54,3430902.37) | 3640548.35 (856167.14,8012528.62) | 15988.53 (3430.93,37770.51) | 45923.11 (10180.82,104663.00) | 1494463.73 (318475.14,3397436.59) | 3594625.24 (843576.73,7906892.03) |
| High SDI | 29449.64 (6629.81,64865.30) | 61892.12 (15113.76,131908.12) | 620262.32 (137654.11,1371665.16) | 1142109.23 (275484.99,2429324.66) | 7679.73 (1679.81,17773.99) | 18179.85 (4257.82,40876.73) | 612582.59 (136205.44,1353322.36) | 1123929.38 (270827.09,2391237.97) |
| High-middle SDI | 20386.63 (4199.16,46932.23) | 49559.18 (11247.02,109612.75) | 477190.62 (95991.69,1107714.65) | 1028031.61 (226138.13,2289283.34) | 4570.03 (912.31,10969.23) | 12323.97 (2659.19,28540.28) | 472620.58 (95085.22,1096789.80) | 1015707.64 (223468.79,2266722.41) |
| Middle SDI | 12527.11 (2767.66,28897.30) | 48932.98 (11229.78,109849.01) | 296614.93 (63689.66,689542.78) | 1047826.8 (237445.14,2385662.87) | 2692.23 (578.93,6409.95) | 11398.83 (2448.23,26650.89) | 293922.70 (63043.41,683143.39) | 1036427.97 (235138.89,2360358.38) |
| Low-middle SDI | 3767.72 (759.67,8775.95) | 14939.97 (3363.04,33465.86) | 89947.58 (17969.49,210361.87) | 336381.71 (73461.95,759593.43) | 806.84 (163.70,1958.76) | 3225.10 (687.14,7525.10) | 89140.74 (17810.32,208462.38) | 333156.60 (72704.02,751487.84) |
| Low SDI | 1095.16 (200.43,2727.10) | 3624.05 (752.72,8309.58) | 25644.72 (4760.50,63969.68) | 84122.18 (17272.26,196759.00) | 231.52 (42.18,595.31) | 772.04 (157.92,1842.67) | 25413.20 (4706.26,63378.11) | 83350.14 (17114.31,194636.54) |
| **Women** | | | | | | | | |
| Global | 17282.05 (3229.81,41144.83) | 54946.59 (10810.53,128856.67) | 370828.23 (68524.76,885559.90) | 1071426.70 (211541.23,2554060.70) | 4167.87 (757.47,10346.28) | 14341.77 (2671.86,34708.83) | 366660.36 (67869.75,875870.95) | 1057084.92 (209246.19,2521423.30) |
| High SDI | 8088.68 (1537.07,19077.58) | 21908.01 (4347.42,50194.66) | 164757.58 (31090.59,388628.27) | 393347.39 (78850.41,903465.71) | 2166.46 (382.46,5253.62) | 6498.09 (1198.93,15585.81) | 162591.12 (30634.14,383024.28) | 386849.30 (77771.60,890221.87) |
| High-middle SDI | 4450.59 (847.36,10710.40) | 13286.66 (2524.38,31661.04) | 96485.01 (17988.66,234980.34) | 264220.98 (50174.13,634365.68) | 976.28 (180.14,2448.73) | 3324.87 (634.05,8162.93) | 95508.74 (17822.56,232699.59) | 260896.11 (49531.13,628166.85) |
| Middle SDI | 3740.55 (717.61,9048.59) | 14668.83 (2839.9,35346.85) | 86037.48 (16284.5,210535.32) | 301896.83 (58214.56,737083.36) | 802.37 (153.27,2031.55) | 3397.89 (638.03,8502) | 85235.11 (16143.75,208539.70) | 298498.94 (57567.40,727075.34) |
| Low-middle SDI | 835.24 (155.44,2010.94) | 4253.48 (873.20,10169.12) | 19598.63 (3673.58,47746.79) | 92872.57 (18925.69,224944.66) | 184.27 (33.67,471.17) | 935.45 (180.05,2373.09) | 19414.36 (3633.34,47330.96) | 91937.12 (18736.02,222970.61) |
| Low SDI | 158.44 (28.82,392.08) | 800.12 (158.58,1895.71) | 3773.77 (686.94,9322.89) | 18499.98 (3663.97,44285.73) | 36.61 (6.32,94.53) | 178.55 (32.70,450.92) | 3737.17 (681.17,9228.58) | 18321.43 (3628.05,43810.35) |
| **Men** | | | | | | | | |
| Global | 49981.68 (8133.5,118885.49) | 124102.19 (21111.53,286268.90) | 1139624.03 (184400.31,2738860.82) | 2569121.65 (432802.98,5909142.51) | 11820.66 (1850.18,28825.55) | 31581.33 (5295.77,75196.23) | 1127803.37 (182260.81,2711764.68) | 2537540.32 (427473.50,5838335.25) |
| High SDI | 21360.96 (3512.83,49580.67) | 39984.11 (6937.3,89358.65) | 455504.74 (73904.08,1067363.88) | 748761.84 (128579.17,1688823.76) | 5513.27 (887.81,13279.38) | 11681.77 (1998.38,27723.55) | 449991.47 (73127.54,1054364.64) | 737080.07 (126574.96,1663775.42) |
| High-middle SDI | 15936.04 (2569.86,38412.96) | 36272.52 (5943.08,83456.93) | 380705.61 (60974.72,926477.51) | 763810.63 (125487.72,1786757.57) | 3593.76 (553.13,9005.68) | 8999.1 (1514.11,21517.48) | 377111.85 (60327.29,917999.12) | 754811.53 (123972.10,1765779.54) |
| Middle SDI | 8786.56 (1368.53,21076.85) | 34264.16 (5565.64,80440.06) | 210577.45 (32314.86,510318.53) | 745929.97 (120559.45,1744906.98) | 1889.86 (290.70,4735.01) | 8000.94 (1274.30,19539.42) | 208687.59 (32024.68,506414.79) | 737929.03 (119327.20,1729004.36) |
| Low-middle SDI | 2932.49 (452.91,7210.27) | 10686.50 (1858.17,24839.72) | 70348.95 (10668.79,172925.68) | 243509.14 (41924.13,567563.60) | 622.57 (93.05,1572.86) | 2289.65 (383.87,5566.15) | 69726.38 (10573.47,171711.88) | 241219.48 (41509.14,563038.18) |
| Low SDI | 936.72 (146.64,2425.41) | 2823.93 (479.71,6916.27) | 21870.95 (3396.07,57456.85) | 65622.2 (10914.79,161946.69) | 194.91 (29.12,530.58) | 593.48 (94.56,1477.92) | 21676.04 (3363.87,56964.61) | 65028.71 (10820.01,160623.15) |

DALYs, Disability-Adjusted Life Years; SDI, Socio-demographic Index; YLDs, Years Lived with Disability; YLLs, Years of Life Lost

**Supplemental Table 2.** The population-attributable fractions of tracheal, bronchus, and lung cancer attributable to high fasting plasma glucose in 1990 and 2019, by sexes and SDI levels.

|  | PAF of Death | | PAF of DALY | | PAF of YLD | | PAF of YLL | |
| --- | --- | --- | --- | --- | --- | --- | --- | --- |
|  | 1990 | 2019 | 1990 | 2019 | 1990 | 2019 | 1990 | 2019 |
| **Both** | | | | | | | | |
| Global | 6.51 (1.45,14.47) | 8.84 (2.05,19.21) | 5.75 (1.27,12.88) | 7.99 (1.83,17.45) | 6.28 (1.40,13.92) | 8.51 (1.98,18.43) | 5.75 (1.27,12.87) | 7.99 (1.83,17.43) |
| High SDI | 7.30 (1.64,16.12) | 10.45 (2.51,22.14) | 6.64 (1.47,14.79) | 9.64 (2.31,20.53) | 7.06 (1.58,15.60) | 9.89 (2.36,21.06) | 6.64 (1.47,14.78) | 9.63 (2.31,20.52) |
| High-middle SDI | 5.73 (1.22,12.95) | 8.05 (1.81,17.72) | 5.03 (1.04,11.57) | 7.27 (1.61,16.08) | 5.43 (1.15,12.34) | 7.64 (1.73,16.74) | 5.03 (1.03,11.57) | 7.27 (1.61,16.07) |
| Middle SDI | 6.25 (1.40,13.95) | 7.99 (1.81,17.64) | 5.39 (1.21,12.23) | 7.22 (1.62,15.89) | 5.85 (1.31,12.99) | 7.55 (1.71,16.55) | 5.38 (1.21,12.22) | 7.22 (1.62,15.88) |
| Low-middle SDI | 5.98 (1.27,13.61) | 8.88 (2.05,19.5) | 5.24 (1.11,12.06) | 7.98 (1.82,17.60) | 5.64 (1.20,12.82) | 8.45 (1.93,18.37) | 5.24 (1.11,12.05) | 7.97 (1.82,17.59) |
| Low SDI | 5.90 (1.13,13.62) | 8.43 (1.86,18.76) | 5.18 (0.99,12.03) | 7.56 (1.67,16.86) | 5.55 (1.06,12.84) | 8.02 (1.80,17.76) | 5.18 (0.99,12.03) | 7.56 (1.67,16.84) |
| **Women** | | | | | | | | |
| Global | 6.36 (1.17,14.90) | 8.35 (1.56,19.18) | 5.56 (1.02,13.11) | 7.46 (1.39,17.24) | 6.09 (1.12,14.30) | 7.99 (1.50,18.59) | 5.56 (1.02,13.10) | 7.45 (1.39,17.22) |
| High SDI | 6.71 (1.24,15.69) | 9.45 (1.82,21.65) | 6.01 (1.11,14.11) | 8.57 (1.65,19.74) | 6.41 (1.19,15.06) | 8.85 (1.73,20.34) | 6.01 (1.11,14.10) | 8.57 (1.65,19.73) |
| High-middle SDI | 5.78 (1.05,13.77) | 7.44 (1.38,17.49) | 4.98 (0.9,11.95) | 6.59 (1.23,15.59) | 5.42 (0.98,12.90) | 6.98 (1.30,16.44) | 4.98 (0.90,11.94) | 6.59 (1.23,15.57) |
| Middle SDI | 6.34 (1.18,14.91) | 7.73 (1.41,17.96) | 5.41 (0.99,12.83) | 6.89 (1.25,16.15) | 5.93 (1.09,14.01) | 7.28 (1.36,17.05) | 5.40 (0.99,12.82) | 6.89 (1.25,16.14) |
| Low-middle SDI | 5.85 (1.09,13.96) | 8.37 (1.59,19.19) | 4.99 (0.92,11.91) | 7.35 (1.38,17.10) | 5.50 (1.01,13.10) | 7.93 (1.53,18.37) | 4.99 (0.92,11.90) | 7.35 (1.38,17.09) |
| Low SDI | 5.10 (0.96,12.12) | 7.68 (1.51,18.03) | 4.36 (0.81,10.41) | 6.74 (1.31,15.91) | 4.82 (0.89,11.48) | 7.29 (1.42,17.16) | 4.36 (0.81,10.40) | 6.73 (1.31,15.90) |
| **Men** | | | | | | | | |
| Global | 6.69 (1.09,15.73) | 9.15 (1.55,20.91) | 5.93 (0.95,14.08) | 8.30 (1.39,19.19) | 6.48 (1.05,15.28) | 8.84 (1.50,20.18) | 5.92 (0.95,14.06) | 8.29 (1.38,19.18) |
| High SDI | 7.65 (1.27,17.66) | 11.1 (1.93,24.8) | 7.01 (1.15,16.46) | 10.34 (1.8,23.35) | 7.46 (1.23,17.31) | 10.60 (1.82,23.99) | 7.01 (1.15,16.45) | 10.33 (1.80,23.34) |
| High-middle SDI | 5.89 (0.95,14.07) | 8.4 (1.41,19.42) | 5.19 (0.83,12.61) | 7.63 (1.27,17.78) | 5.60 (0.90,13.45) | 8.01 (1.34,18.41) | 5.19 (0.83,12.61) | 7.62 (1.27,17.77) |
| Middle SDI | 6.31 (1.02,14.95) | 8.17 (1.35,18.87) | 5.45 (0.88,13.09) | 7.42 (1.22,17.34) | 5.90 (0.95,14.11) | 7.72 (1.27,17.96) | 5.45 (0.88,13.08) | 7.41 (1.22,17.34) |
| Low-middle SDI | 6.06 (0.96,14.55) | 9.15 (1.53,21.08) | 5.34 (0.84,12.84) | 8.29 (1.39,19.16) | 5.72 (0.90,13.75) | 8.72 (1.46,20.16) | 5.34 (0.84,12.83) | 8.28 (1.38,19.15) |
| Low SDI | 6.10 (0.97,14.60) | 8.7 (1.46,20.24) | 5.38 (0.85,12.98) | 7.86 (1.31,18.35) | 5.74 (0.92,13.71) | 8.30 (1.39,19.25) | 5.37 (0.85,12.98) | 7.85 (1.31,18.35) |

DALYs, Disability-Adjusted Life Years; PAF, population-attributable fractions; SDI, Socio-demographic Index; YLDs, Years Lived with Disability; YLLs, Years of Life Lost


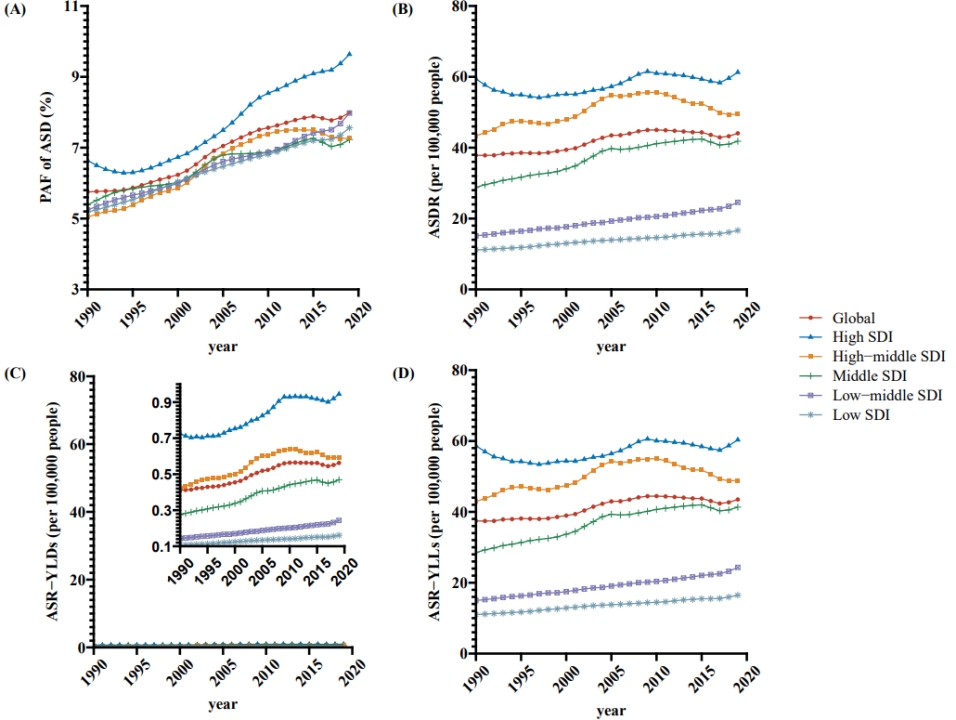


**Supplemental Figure 1.** The changing trend of TBL cancer burden attributable to HFPG globally and in SDI regions, from 1990 to 2019. (A) PAF; (B) DALYs; (C) YLDs; (D) YLLs. DALYs, Disability-Adjusted Life Years; HFPG, High Fasting Plasma Glucose; PAF, Population Attributable Fraction; SDI, Socio-demographic Index; YLD, Years Lived with Disability; YLL, Years of Life Lost.
